# Cytologic features of microcystic adnexal carcinoma

**DOI:** 10.4103/1742-6413.77285

**Published:** 2011-03-03

**Authors:** Sasis Sirikanjanapong, Andrew W. Seymour, Bijal Amin

**Affiliations:** Depertment of Cytopathology, Montefiore Medical Center Albert Einstein College of Medicine, 111 210 Street Bronx, NY 10467, USA; 1 Department of Dermatopathology, Montefiore Medical Center Albert Einstein College of Medicine, 111 210 Street Bronx, NY 10467, USA

**Keywords:** Dermatology, microcystic adnexal carcinoma, skin neoplasm

## Abstract

Microcystic adnexal carcinoma (MAC) is an uncommon skin neoplasm with a predilection location around the lips. It is characterized by cords and nests of neoplastic cells forming ductular or glandular structures that are embedded in dense collagenous stroma. An eighty-seven year old Caucasian female patient presented with a painless, slowly enlarging mass measuring 3.3 × 2.7 × 1.0 cm on the lower lip for approximately 6 months. The patient underwent 2 fine needle aspiration biopsies (FNAs). Smears made from both FNAs demonstrated similar features including low cellular smears, three dimensional cell clusters forming a glandular structure, round to oval cells with high N:C ratio, occasional cytoplasmic lumens, without distinct hyperchromasia, focal inconspicuous nucleoli, smooth regular nuclear membranes, abundant naked nuclei, occasional squamoid cells and focal acellular stromal fragments in the background. The cytologic differential diagnosis included skin adnexal carcinoma and low grade mucoepidermoid carcinoma arising in the minor salivary gland. The mass was subsequently excised. The diagnosis of microcystic adnexal carcinoma was made. We report cytologic features of MAC and also suggest that MAC can possibly be diagnosed by FNA with the appropriate clinical vignette and immunohistochemical profile..

## INTRODUCTION

Microcystic adnexal carcinoma (MAC) is an unusual skin neoplasm. It was first described as a distinct entity by Goldstein *et al*. in 1982.[1] The authors reported six cases which demonstrated similar features, including islands of basaloid keratinocytes with occasional horn cysts and abortive hair follicles in a desmoplastic stroma. Similar cases might have been previously reported as malignant syringoma. A total of 223 cases of MAC were identified by Surveillance, Epidemiology, and End results registry 1973–2004. They were able to identify the predilection site of this entity for the head and neck area (74%).[2]

## CASE REPORT

**Figure 1:**
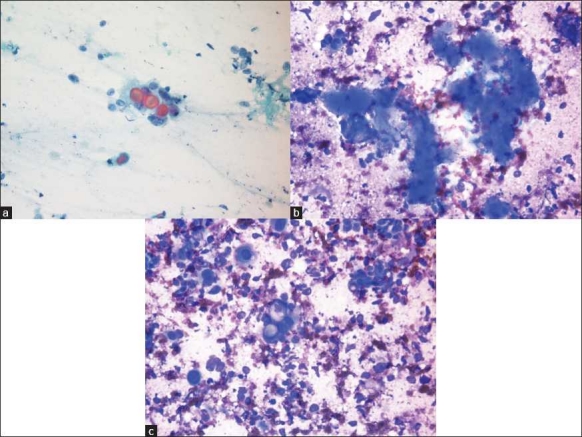
a) A three dimensional cluster of epithelial cells with low-grade atypia form a glandular structure with intraluminal content. The cells demonstrate high N: C ratio (Pap ×400); b) Occasional basophilic stromal fragments are identified (Diff Quik ×400); c) A small cluster of neoplastic cells with cytoplasmic vacuoles with several stripped nuclei in the background are identified (Diff Quik ×400)

**Figure 2:**
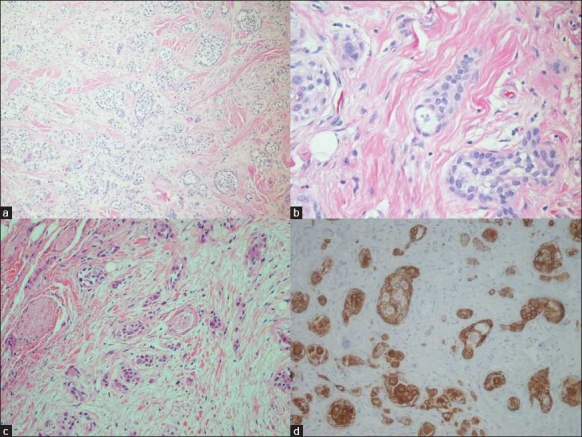
a) The neoplasm forming small islands and microcysts infiltrates underlying skeletal muscles (H and E, ×100); b) The neoplastic cells demonstrate inconspicuous nucleoli and low-grade atypia corresponding to the FNA findings (H and E, ×400); c) Perineural invasion is identified (H and E, ×400); d) The neoplastic cells are immunohistochemically positive for AE1/AE3 (×400) ConsentWritten informed consent was obtained from the patient for publication of this case report and any accompanying images. A copy of the written consent is available and authors take responsibility to maintain relevant documentation in this respect.

An 87-year-old Caucasian female patient presented to the ear nose and throat (ENT) clinic with a painless, slowly enlarging mass on the lower lip for approximately 6 months. She denied a history of previous malignancies. Physical examination revealed a firm, flesh-colored, indurated thick plaque measuring 3.3 × 2.7 × 1.0 cm, located 0.5 cm away from the lower vermilion border. There were no epidermal changes. No lymphadenopathy was noted. The patient underwent two fine-needle aspiration (FNA) biopsies, each one month apart. Both FNAs revealed similar findings, including paucicellular smears, round tight three dimensional cell clusters, round to oval cells with high N: C ratio, occasional cytoplasmic lumens, without distinct hyperchromasia, focal inconspicuous nucleoli, smooth regular nuclear membranes, abundant naked nuclei, and focal acellular stromal fragments in the background. Occasional squamoid cells were identified [Figure 1a–c]. The diagnoses were reported as “Epithelial neoplasm, favor adnexal tumor.” The differential diagnosis included salivary gland neoplasm, especially a low-grade mucoepidermoid carcinoma. The patient received a wide excision. On histopathological examination, the tumor consisted of islands and cords of cells with mild atypia, as well as occasional tadpole-like ductular formation and microcysts embedded in a dense desmoplastic stroma. The neoplasm infiltrated the underlying skeletal muscle. Focal horn cysts were present, as well as perineural infiltration. The tumor extended close to the deep margin [Figure 2a–c]. Periodic acid-Schiff diastase (PAS-D) and mucin stain failed to demonstrate mucin deposit in the lumen of the ductular structure. The tumor cells were immunohistochemically reactive to AE1/AE3, but nonreactive to S-100 and BerEP4 [Figure 2d]. The diagnosis of MAC was made.

## DISCUSSION

MAC is an uncommon, locally aggressive adnexal neoplasm. The tumor occurs mostly on the face, especially the lips.[3] MAC has also been reported to occur on perianal areas.[4]

After conducting an extensive literature search, we found that the cytologic features of MAC by FNA have been described once by Orell *et al*. The authors reviewed the case that had been misdiagnosed as infiltrating basal cell carcinoma on both FNA and surgical biopsy. They described the tight clusters of basaloid cells with microtubular structure and squamous differentiation.[5] Also, only a handful of FNA diagnoses of benign adnexal neoplasms have been described.[6,7] Histologically, the differential diagnosis of MAC includes desmoplastic trichoepithelioma (DT) and morpheaform basal cell carcinoma (MBCC). All have similar histologic features, including ductule-like structures, round cells with high N : C ratio, and dense desmoplastic stroma. Unfortunately, FNA characteristics of both entities have not been described. However, the cytologic features of basal cell carcinoma have been well described. They include large tight clusters of crowded atypical basaloid cells without the palisading pattern that is normally seen histologically. In contrast to conventional BCC, morpheaform BCC is histologically less cellular and tends to form small islands and duct-like structures. Therefore, FNA from morpheaform BCC is expected to be less cellular and may not yield typical crowded basaloid clusters.

Ferrara *et al*. reported FNA findings of low-grade adenosquamous carcinoma of the breast (LASCB) which is a rare entity.[8] The histologic characteristics of LASCB include “tadpole”-shaped ductal formation composed of epithelial cells with low-grade atypia, bland stroma, and epidermoid cells. And these findings are very similar to those of MAC. Theoretically, the FNA findings of LASCB should be similar to those of MAC as well. Ferrara *et al*. described a moderately cellular smear with prevalent small clusters of overlapping ductal cells. The epithelial cells are small and monotonous. They exhibit inconspicuous nucleoli. Additionally, they identified a number of fibroblast-like spindle cells singly and in clusters.

We noticed some similarities among the cytologic features of MAC in this current study, the case that was reviewed and described by Orell *et al*., and those of LASCB described by Ferrara *et al*. These include dual population of cell, which include both epithelial and stromal cells. Ductular formation is observed with round cells showing low-grade atypia. We believe that there may possibly be enough morphologic evidence to establish diagnostic criteria for MAC.

We suggest that with the appropriate clinical vignette, the diagnosis of MAC could be made. Table 1 describes the diagnostic features of MAC that we would like to propose.

**Table 1:** Summary of cytologic and clinical features of MAC

*Diagnostic features of MAC on FNA*
Three dimensional clusters of epithelial cells that form glandular/ductular structures
Low-grade atypia
Stromal fragments
Naked nuclei
Squamoid cells
Location of the lesion on the face, especially around the lips
Slowly enlarging mass with firm to hard consistency

MAC - Microcystic adnexal carcinoma; FNA - fine-needle aspiration

There have been a number of studies of immunohistochemistry on MAC and its mimickers, including DT and MBCC.[9,10] Cytokeratin (CK) 15 can be helpful in distinguishing MAC from BCC. Hoang *et al*. demonstrated that 92% of MAC and 100% of trichoepithelioma were immunohistochemically reactive to CK15, whereas 0% of BCC showed immunoreactivity to CK15.[10] They also reported that 38% of MAC, 57% of DT, and 100% of MBCC were positive for BerEP4.[10] Another similar study by Smith *et al*. reported that all of their cases of MAC, MBCC, and DT were all negative for BerEP4.[9] Our current case is also negative for BerEP4. Smith *et al*. also demonstrated that a small percentage of tumor cells in two of ten MAC cases were positive for P53, whereas 0% of DT were positive for P53.[9] Statistically, none of the immunohistochemical markers performed in this study was proven to be useful for distinguishing between MAC and DT.

Although it is difficult to exclude the possibility of DT when facing the cytologic challenge of MAC despite the aid of immunohistochemical studies, it is still possible to distinguish MAC from DT by using the clinical manifestations. DT commonly presents on the cheek and forehead, in contrast to MAC which mainly occurs around the lips. The clinical appearance of DT is also different from MAC. DT typically manifests as annular dermal papules or plaques.[11] MAC usually presents as ill-defined round papules or plaques without central depression; therefore, the clinical context including the location and appearance of the lesion is essential for making the diagnosis.

The treatment options for MAC include an excision, Mohs surgery, and radiotherapy. Although Mohs surgery has been reported to be the preferred method,[12] standard treatment is still unclear.

In conclusion, we report the cytologic features of MAC that have been described in the literature only once. We also suggest that MAC can be diagnosed by FNA with the appropriate clinical vignette and immunohistochemical profile. But the diagnosis should be confirmed by a conventional biopsy due to limited studies on the cytologic diagnosis of MAC.

## COMPETING INTEREST STATEMENT BY ALL AUTHORS

The authors declare that they have no competing interests

## AUTHORSHIP STATEMENT BY ALL AUTHORS

Each author acknowledges that this final version was read and approved. All authors of this article declare that we qualify for authorship as defnied by ICMJE http://www.icmje.org/#author. Each author has participated sufficiently in the work and take public responsibility for appropriate portions of the content of this article.

## ETHICS STATEMENT BY ALL AUTHORS

Our institution does not require approval from the Insti-tutional Review Board for a case report without identifiers.

## EDITORIAL / PEER-REVIEW STATEMENT

To ensure integrity and highest quality of CytoJournal publications, the review process of this manuscript was conducted under a double blind model(authors are blinded for reviewers and reviewers are blinded for authors)through automatic online system.

## References

[1] Goldstein DJ, Barr RJ, Santa Cruz DJ (1982). Microcystic Adnexal Carcinoma: A Distinct Clinicopathologic Entity. Cancer.

[2] Yu JB, Blitzblau RC, Patel SC, Decker RH, Wilson LD (2010). Surveillance, Epidemiology, and End Results (SEER) Database Analysis of Microcystic Adnexal Carcinoma (Sclerosing Sweat Duct Carcinoma) of the Skin. Am J Clin Oncol.

[3] Bier-Laning CM, Hom DB, Gapany M, Manivel JC, Duvall AJ (1995). Microcystic adnexal carcinoma: Management based on long-term follow- up. Laryngoscpoe.

[4] Murata S, Fujita S, Sugihara K, Akasu T, Moriya Y, Nakanishi Y (1997). Sclerosing Sweat Duct Carcinoma in the Peri-anal Skin: A case report. Jpn J Clin Oncol.

[5] Orell SR, Sterrett GF, Whitaker D, Orell SR (2005). Skin and Subcutis. Fine needle aspiration cytology.

[6] Lemos MM, Kindblom LG, Meis-Kindblom JM, Ryd W, Willen H (2001). Fine-needle aspiration features of pilomatrixoma. Cancer.

[7] Dubb M, Michelow P (2009). Cytologic features of hidradenoma in fine needle aspiration biopsies. Acta Cytol.

[8] Ferrara G, Nappi O, Wick MR (1999). Fine-Needle Aspiration Cytology and Immuohistoolgy of Low-Grade Adenosquamous carcinoma of the Breast. Diagn Cytopathol.

[9] Smith K, Williams J, Corbett D, Skelton H (2001). Micorcystic Adnexal Carcinoma: An Immunohitochemical Study Including Markers of Proliferation and Apoptosis. Am J Surg Pathol.

[10] Hoang MP, Dresser KA, Kapur P, High WA, Mahalingam M (2008). Microcystic Adnexal Carcinoma: An immunohistochemical Reapraisal. Mod Pathol.

[11] Brownstein MH, Shapiro L (1977). Desmoplastic Trichoepithelioma. Cancer.

[12] Metze D, Grunert F, Neumaier M, Bhardwaj R, Amann U, Wagener C (1996). Neoplasm with sweat gland differentiation expressing various glycoproteins of CEA family. J Cutaneous Pathology.

